# Analyzing the Geometry and Dynamics of Viral Structures: A Review of Computational Approaches Based on Alpha Shape Theory, Normal Mode Analysis, and Poisson–Boltzmann Theories

**DOI:** 10.3390/v15061366

**Published:** 2023-06-13

**Authors:** Yin-Chen Hsieh, Marc Delarue, Henri Orland, Patrice Koehl

**Affiliations:** 1 Institute for Arctic and Marine Biology, Department of Biosciences, Fisheries, and Economics, UiT The Arctic University of Norway, 9037 Tromso, Norway; hsieh.y.chen@uit.no; 2 Institut Pasteur, Université Paris-Cité and CNRS, UMR 3528, Unité Architecture et Dynamique des Macromolécules Biologiques, 75015 Paris, France; delarue@pasteur.fr; 3 Institut de Physique Théorique, CEA, CNRS, Université Paris-Saclay, 91191 Gif-sur-Yvette, France; henri.orland@cea.fr; 4 Department of Computer Science, University of California, Davis, CA 95616, USA

**Keywords:** virus structure, alpha shapes, normal modes, electrostatics

## Abstract

The current SARS-CoV-2 pandemic highlights our fragility when we are exposed to emergent viruses either directly or through zoonotic diseases. Fortunately, our knowledge of the biology of those viruses is improving. In particular, we have more and more structural information on virions, i.e., the infective form of a virus that includes its genomic material and surrounding protective capsid, and on their gene products. It is important to have methods that enable the analyses of structural information on such large macromolecular systems. We review some of those methods in this paper. We focus on understanding the geometry of virions and viral structural proteins, their dynamics, and their energetics, with the ambition that this understanding can help design antiviral agents. We discuss those methods in light of the specificities of those structures, mainly that they are huge. We focus on three of our own methods based on the alpha shape theory for computing geometry, normal mode analyses to study dynamics, and modified Poisson–Boltzmann theories to study the organization of ions and co-solvent and solvent molecules around biomacromolecules. The corresponding software has computing times that are compatible with the use of regular desktop computers. We show examples of their applications on some outer shells and structural proteins of the West Nile Virus.

## 1. Introduction

The recent COVID-19 pandemic has highlighted how fragile human health is as we continue to be exposed to emerging pathogens for which we do not always have available treatments. In addition, as many experts are warning us, such exposures are likely to increase as a result of climate change and global warming (see for example the recent reviews on the impact of climate change on eye diseases [1], on allergy epidemics [2], and on zoonotic diseases [3,4]). Pandemics are costly economically [5,6]; more worrisome, they have a high level of mortality. As of today, for example, COVID-19 is estimated to be responsible for more than 14.5 million excess deaths [7]. As severe as this seems to be, other recent pandemics have been deadlier: in the first half of the 20th century, the Spanish influenza pandemic was responsible for at least 50 million deaths [8].

Infectious diseases are caused by pathogens, the most common of them being viruses, bacteria, fungi, and parasites. While all may lead to serious diseases and pandemics, viruses are the most common (for instance, 5% to 20% of Americans are exposed to the influenza virus every year). The consequences of viral infection are varied, from minor (common cold) to major (flu—influenza, COVID-19—SARS-CoV-2, immune deficiencies—HIV, among others). The recent COVID-19 pandemic brought wide awareness to the need for prevention, treatments, and cures to help protect populations. All efforts in those directions require knowledge of the epidemiology associated with the virus of concern and of its biology. Again, the COVID-19 epidemic is a good example of efforts and successes of the scientific community to analyze and combat the virus responsible for this disease, SARS-CoV-2. Studies on its pathogenesis [9,10], its evolution with consequences on its biology [11,12,13], and on our innate immunity against it [14], complemented with studies on its structural biology [15,16,17,18,19,20], have led to major vaccine developments that have ultimately curbed the pandemic [21] (a good indicator of the urgency to find cures for COVID-19 and the corresponding response of the community is found in the fact that it is estimated that 10,000 articles were published every month on a topic associated with COVID-19 in 2020 [22,23], to the point that it is changing the landscape of scientific publishing [24]). In this review, we are concerned with the data associated with the structural biology of viruses, as well as the models and methodologies that enable us to analyze those data and ultimately with the tools that help identifying antiviral drugs.

A virus particle, known also as a virion, consists of a nucleic acid (DNA or RNA) that is surrounded with a protective coat referred to as a capsid. The first structural studies of such viral particles were possibly conducted by Bernal and Fankuchen in 1938, using crystallographic data to show that the Tomato Bushy Stunt Virus (TBSV) crystallizes in a body-centered cell structure [25]. Their analyses were based on powder photographs instead of single crystals. It was only in the 1970s that crystallographic experimental techniques and associated computing methods enabled atomic-level resolution of whole viral particles. One of the first of such structures was of TBSV at 5.5 Å resolution [26]. This structure was deposited in the then-recently assembled Protein Data Bank (PDB) [27,28]. Since then, the number of virion structures in the PDB as well as the number of proteins expressed by viral genomes has increased drastically (see for example [29] for a review of icosahedral virus structures in the PDB [30]). To help virologists with exploring those structures, Reddy and colleagues developed a virus particle explorer, VIPER [31,32,33,34,35]. There are five main geometries for virions: helical (such as tobacco mosaic virus and inovirus), icosahedral (see [30] for a full review), prolate (often found in bacteriophage), enveloped (in which the capsid is enveloped by a modified form of a cell membrane, found in influenza viruses, HIV, and coronaviruses, for example), and complex (i.e., different from the other types). Similarly, the proteins of the envelope of those viruses adopt many different folds [36,37,38].

The PDB and VIPERDB [34], the database associated with VIPER, have proven to be essential resources for understanding virions and viral protein structures. The experimental resolutions of such structures remain, however, a significantly time-consuming and expensive task. There is hope, however, that computational techniques can complement experimentation significantly and accurately, as demonstrated with the recent successes of AlphaFold [39] and its successor AlphaFold2 [40]. Those programs, based on artificial intelligence, were designed by the company DeepMind to predict the conformation of a protein from its sequence only. They are based on deep learning techniques to predict those structures at near experimental scale resolution. They are inspiring biologists to rethink the way they study the function and evolution of proteins [41,42,43]. There are, however, limitations to AlphaFold/AlphaFold2 [43]. AlphaFold is designed to generate the most probable conformation of a protein structure. As such, it cannot provide information on the ensemble of conformations that may exist for intrinsically disordered proteins. In addition, the solution it provides is static and as such, does not capture essential dynamics of proteins, such as allostery. Finally, it ignores the fact that many proteins function as complexes with other proteins, nucleic acids, or ligands. As such ligands include drug-like molecules, its impact on drug design remains limited [44,45]. While we can be optimistic with respect to the opportunities of artificial intelligence to establish itself as the solution to analyzing protein dynamics and to designing efficient drugs against protein targets, current efforts still rely on biophysics.

Understanding the complex geometry of virions, their assembly, and dynamics has always been a topic of great interest mathematical biology and biophysics, defining subfields in both disciplines, mathematical virology and physical virology (see for example [46,47,48]). Virus structures are seem as mathematical puzzles [49,50,51,52] whose solutions may lead to a novel understanding of protein assembly and ultimately to novel packaging options for drug delivery [53,54]. Understanding how genome size, electrostatics of interactions with the nucleic acids forming this genome [55,56,57], stiffness [58], and dynamics [59] affect how viral structures form within the host cells is essential for the development of drugs that would disrupt virus assembly [19]. With the advent of new hardware and better algorithms, it is now possible to study the dynamics of viral particles using all atom molecular dynamics simulations, a first step in understanding how they adapt to different environments as well as to immunological factors, such as the binding of an antibody [58,60,61,62,63,64]. All those studies rely on either coarse grained models and/or the availability of significant computing power. Understanding viral geometries, dynamics, and energetics at the atomic level with a computing power manageable to a virologist are essential steps for them to understand viral biology, and more importantly, to understand how to inhibit viruses, i.e., to develop antiviral drugs.

In this paper, we review theories and methodologies we have developed that enable the analyses at these three levels for viral structures. We discuss those methodologies in light of the specificities of those structures, mainly that they are huge and may include millions of atoms. We focus on methods that can analyze such structures within computing times that are compatible with the use of regular desktop computers. We use the structure of the envelope of flaviviruses to illustrate those methods. Flaviviruses belongs to the flaviviridae family. Those viruses, such as yellow fever virus (YFV), Dengue virus (DENV), West Nile virus (WNV), and ZIKA virus, continue to pose a major threat to human health [65]. Most of the flaviviruses are enveloped single-stranded RNA viruses with icosahedral symmetry. Their envelopes are large (usually 180 copies of two proteins, the E protein and the M protein), making them good tests of the efficiencies of the methodologies described in the review.

None of the methods presented in the paper are novel: they all come from previous studies from the authors. As such, this paper is deemed to be a review. Its novelty, however, lies in the fact that it describes a comprehensive sets of tools for studying the geometry and dynamics of very large molecular systems, such as viral particles. In addition, it shows novel applications of those methods for studying the dynamics of flaviviruses, mostly West Nile viruses. The review includes three sections, corresponding to studying the geometry (Section 2), the dynamics (Section 3), and the energetics (Section 4) of virus structures. Each section comprises a motivation with a review of current work, a presentation of the methodology we propose, and examples of application of this methodology.

## 2. The Geometry of Viral Structures

### 2.1. Motivation and Background

Biochemists have always worked under the assumption that shape defines function. As a consequence, as early as the early 1900s, they have used models to analyze the impact of structure on chemical reactivity. They have subsequently invested in determining the structures of important biomolecules experimentally at atomic resolution. The result of such research describes the biomolecule from the positions of each one of its atoms. The shape of the molecule can then be derived using a space-filling model, where the atoms are represented by balls whose centers are the experimentally derived positions of the atoms and whose radii are proportional to their van der Waals radii [66,67]. Properties of the molecule are then expressed in terms of properties of the union of those balls, leading to geometric modeling of a protein structure:

(i) *Characterizing molecular environments:* the interaction between a molecule and its environment is quantified through the (exposed) surface area and/or volume of the union of balls [68,69,70,71].
(ii) *Evaluate the hydrophobicity of a molecule*. The most common use of molecular shape is the quantification of the hydrophobic effect. Eisenberg and McLachlan, for example [68], introduced the concept of a solvation free energy for large biomolecules, computed as a weighted sum of the accessible areas of all their atoms *i*. This solvation-free energy is a mean force potential that quantifies the energy that is required to solvate a molecule. Its nonpolar contribution is evaluated from geometric measures of the molecule, including surface area [68], volume [72], or even the curvature of the surface area in the so-called morphometric model [73].
(iii) *Identifying pockets and cavities in molecules:* detecting and measuring internal cavities of biomolecules is often performed as a first step for drug design as those cavities map to putative binding sites.

Lee and Richards pioneered the computation of the surface area of a biomolecule by sampling the surface with a set of parallel planes (two-dimensional sections) [74]. Since then, many methods for measuring biomolecules have been proposed, based on Monte Carlo integrations [75,76,77,78,79], on analytical approximations [80,81,82,83,84,85], or even on comprehensive analytical methods [86,87,88,89]. Similarly, many methods have been proposed to detect pockets and cavities in proteins, based on representation of the molecule of interest on a 3D grid [79,90,91,92,93], or scanning the surface with a probe [94,95,96,97].

The alpha shape theory is a comprehensive method for measuring unions of balls using the Voronoi decomposition of the union [89,98,99,100,101,102,103,104,105,106]. It is fast, robust, and amenable to the study of large biomolecular systems [103,106,107]. In the following, we briefly introduce the theory and show applications for measuring the envelope of the WNV.

### 2.2. Methodology

Consider a set of *N* three-dimensional balls, $B_{i}$, that may overlap. The principle of inclusion–exclusion enables the computation of any geometric measure of the union of the $B_{i}$. Such a measure is then expressed as a sum of alternating signs of the measures of the intersections of the $B_{i}$. This approach, however, is of limited interest unless two issues are solved. First, the number of terms in the sum needs to be significantly reduced, as the total number of possible intersections of $B_{i}$ s $2^{N}-1$, leading to exponential running time. This reduction needs to be exact, i.e., the resulting reduced sum needs to give the same result as the full summation. Second, analytical formulas are needed to compute the measures of these intersections of balls. The next two subsections provide solutions to these two issues. Note that more comprehensive presentations of those solutions can be found in References [100,103,106].

#### 2.2.1. Voronoi Decompositions and Dual Complexes

**Figure 1:**
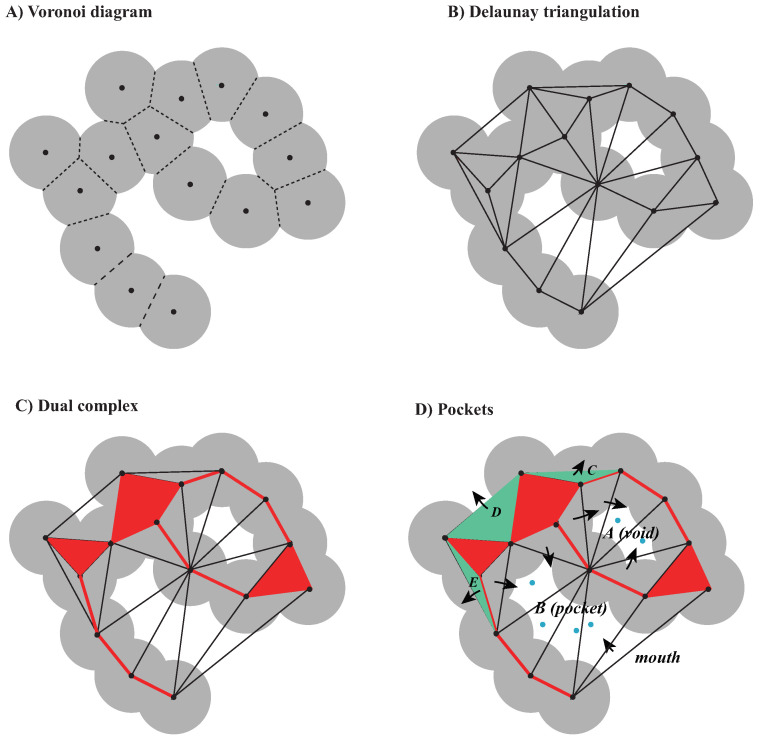
Voronoi decomposition, Delaunay triangulation, dual complex, and pockets of a union of disks. (**A**) Given a finite set of disks, the Voronoi diagram corresponds to a decomposition of the whole plane into regions, one for each disk, such that any point that belongs to the region corresponding to disk $D_{i}$ is closer to that disk than to any other disk (see text for details). In the graphics, we have restricted the Voronoi diagram to the region covered by the disks. (**B**) The Delaunay triangulation is the dual of the Voronoi diagram that is constructed by defining edges between disk centers of neighboring Voronoi regions. (**C**) The dual complex is a subset of the Delaunay triangulation, limited to the edges and triangles (red), whose corresponding Voronoi regions fully intersect within the union of disks. (**D**) All triangles in the Delaunay complex that do not belong to the dual complex are referred to as empty. Acute empty triangles (identified with large blue dots at their orthocenter) contain their orthocenters: they correspond to sinks. The obtuse empty triangles either flow to these acute triangles or to the outside, referred to as “infinity” (those triangles are colored in green). Triangles C, D, and E, for example, flow to infinity: they do not define pockets. The remaining triangles can be partitioned into two groups: region A is completely surrounded by the union of disks and therefore defines a void, while region B is connected to the outside by one mouth, and is referred to as a pocket.

One ball $B_{i}$ in the finite set of balls defining the space filling diagram of a molecule is characterized by its center $z_{i}$ and radius $r_{i}$. We call $S_{i}$ the sphere that is at the boundary of $B_{i}$. We define the *power distance* between a point *x* and a ball $B_{i}$ as $\pi_{i}\left(x\right)={\left|x-z_{i}\right|}^{2}-r_{i}^{2}$. The *Voronoi cell* associated with the ball $B_{i}$ consists of all points *x* that are at least as close to $B_{i}$ as to any other ball: $V_{i}=\left\{x\in R^{3}|\pi_{i}\left(x\right)\leq \pi_{j}\left(x\right)\right\}$. The collection of all Voronoi cells $V_{i}$ form the *Voronoi diagram* of the balls. The intersection of the Voronoi diagram with the union of balls decomposes this union into convex regions, as shown in Figure 1A.

The *Delaunay triangulation* is the dual of the Voronoi diagram [89]. A 2D version of the Delaunay triangulation is illustrated in Figure 1B.

We limit the construction of Delaunay triangulation to within the union of balls. In other words, we draw a dual edge between the two vertices, $z_{i}$ and $z_{j}$, only if $B_{i}\;\cap \;V_{i}$ and $B_{j}\;\cap \;V_{j}$ share a common face, and similarly for triangles and tetrahedra. The result is a subcomplex of the Delaunay triangulation, which is referred to as the *dual complex* of the set of balls (see Figure 1C). The dual complex of the union of balls allows us to apply the inclusion–exclusion formula based on intersections of up to four balls only.

#### 2.2.2. Area and Volume Formulas

Let *K* be the dual complex. A simplex, *s*, in *K* can be seen as a collection of balls: one ball $B_{i}$ if it is a vertex $s_{i}$, two balls $B_{i}$ and $B_{j}$ if it is the edge $s_{ij}$ between their centers, three balls $B_{i}$, $B_{j}$, and $B_{k}$ if it is the triangle $s_{ijk}$ built from their centers, and finally four balls $B_{i}$, $B_{j}$, $B_{k}$, $B_{l}$ if it is the associated tetrahedron between their centers $s_{ijkl}$. As proven in [89], the inclusion–exclusion formula that corresponds to the dual complex gives the correct volume and surface area of a union of balls. Then:

(1) $$\begin{array}{ccc} A_{i} & = & A_{i}-\sum_{j|s_{ij}\in K}A_{i;j}+\sum_{\left(j,k\right)|s_{ijk}\in K}A_{i;jk}-\sum_{\left(j,k,l\right)|s_{ijkl}\in K}A_{i;jkl}, \end{array}$$

(2) $$\begin{array}{ccc} V_{i} & = & V_{i}-\sum_{j|s_{ij}\in K}V_{i;j}+\sum_{\left(j,k\right)|s_{ijk}\in K}V_{i;jk}-\sum_{\left(j,k,l\right)|s_{ijkl}\in K}V_{i;jkl}. \end{array}$$

where $V_{i}$ is the volume of the ball $B_{i}$, $V_{i;j}$ is the contribution of $B_{i}$ to the volume of the intersection of the balls $B_{i}$ and $B_{j}$, etc. Similar definitions are used for the surface areas A.

Proofs of Equations (1) and (2) and additional formula for the values of the different V and A are provided in [89,98,99,103].

#### 2.2.3. Voids and Pockets

References [100,103,108] provide full descriptions of how to detect and measure pockets in a union of balls using the alpha shape theory. Here, we just present the basic concepts. A pocket is connected to the notion of a continuous flow field defined on the Delaunay triangulation of the balls. Let *T* be the set of tetrahedra in the Delaunay triangulation and $T_{\infty }=T⋃\tau_{\infty }$, where $\tau_{\infty }$ is a dummy element representing the complement of the triangulation in $R^{3}$. We define a flow relation “≺” on *T*, such that τ≺σ means:

(i) τ and σ share a common triangle Δ;
(ii) The interior of τ and the orthogonal center $z_{\tau }$ of τ lie on different sides of the plane defined by Δ.

where the orthogonal center $z_{\tau }$ is the center of the smallest ball that is orthogonal to all four balls, whose centers are the vertices of τ.

If τ≺σ, τ is a predecessor of σ and σ is a successor of τ. σ∈T is a sink if it has no successors; in other words, a tetrahedron is a sink if and only if it contains its orthogonal center. Sinks are important since they are responsible for the formation of voids: if *H* is a void of the union of balls, then at least one tetrahedron in *H* is a sink.

By definition, pockets consist of the Delaunay tetrahedra that do not belong to the dual complex *K* and are not predecessors of $\tau_{\infty }$. The only type of pockets without connection to the outside are the voids. All other pockets connect to the outside at one or more places, called a *mouth*. Figure 1D illustrates these concepts.

The surface area and volume of a pocket are easily computed using simplified inclusion–exclusion formulas.

### 2.3. Examples

**Figure 2:**
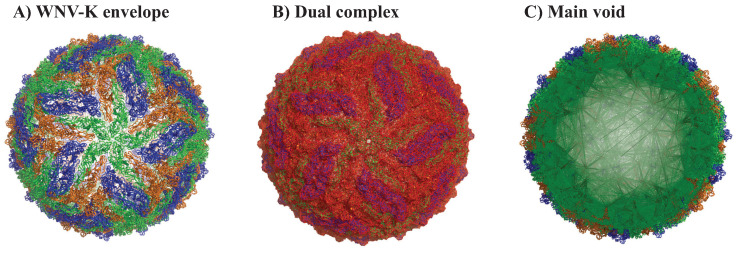
The geometry of the WNV-Kunjin virus. (**A**) Cartoon representation of the mature form of the outer shell of the Kunjin virus (a subtype of WNV) (PDB file 7KVA). The outer shell includes 180 copies of both the E protein and the M protein. The three E proteins from each asymmetric unit are colored green, orange, and blue, respectively. (**B**) The dual complex corresponding to the envelope of the Kunjin virus is shown in red, over the structure of the virus. The simplices of this complex define all the terms of the inclusion–exclusion formula needed to compute the volume and surface area of the virus (**C**) Cross section of the empty envelope of the Kunjin virus, showing in green the main pocket identified by UnionBall. All three panels were generated using Pymol [111].

We illustrate the geometric analysis described above on the structure of the Kunjin variant of the West Nile Virus (WNV-K). WNV is a member of the *flaviviridae* family. It shares a common structural fold with other viruses from the same family, such as Dengue and Zika. All the flaviviruses have their genomic material packaged by a capsid assembled from viral C proteins, forming the nucleocapsid. This nucleocapsid is surrounded by an envelope. This envelope consists of a lipid bilayer membrane, itself covered by an outer shell of proteins, the M protein that is anchored in the lipid membrane, and the E protein. The outer shell has icosahedral symmetry. It is formed of 60 asymmetrical units, with each unit containing three copies of the E (i.e., envelope) protein and three copies of the M (i.e., membrane) protein. The high resolution structure of several WNV viruses are available in the Protein Data Bank. Note that most structural information available only includes the outer shell, as it is difficult to observe the lipid bilayer membrane and the nucleocapsid, although recently, a cryo-EM model of the capsid of immature Zika virus was described [109]. Here, we focus on the structure of the Kunjin virus, a subtype of WNV endemic to Oceania. The all-atom, high resolution structure (3.1 Å) derived from Cryo Electron Microscopy (cryo-EM) of the outer shell of the virus is available in the PDB with the identifier 7KVA [110]. A cartoon representation of this structure is given in Figure 2A. Each asymmetrical unit contains 3 E proteins and 3 M proteins, with a total of 1728 residues (3×501 E protein residues and 3×75 M protein residues) that include 12,835 atoms. The full envelope includes 60 copies of this unit, for a total of 103,680 residues and 770,100 atoms.

#### 2.3.1. Full Viral Envelope

We analyze the geometry of the WNV-K virus outer shell from the PDB file 7KVA using our program UnionBall [103]. It takes a total of 10 s on an AMD Threadripper multicore CPUs running at 2.2 GHz, with 32 cores (64 threads) to fully characterize its outer shell, i.e., to compute the Delaunay triangulation, extract the dual complex, and find the pockets for the union $U_{WNV}$ of 770,100 balls that represents this outer shell. Note that the Delaunay computation is the dominant part of the overall computing cost, taking 5.3 s. As computing the Delaunay is mostly sequential, the whole calculation does not benefit from the multiple cores. Each ball in $U_{WNV}$ is assigned a radius set to the vdW radius of the corresponding atom, to which we add 3 Å (approximately twice the radius of a water molecule). The Delaunay triangulation of the union of balls contains 5,217,324 tetrahedra, while the dual complex contains 4,718,495 tetrahedra, 9,635,746 triangles, and 5,683,487 edges. This dual complex is represented in red in Figure 2B. It includes one large void (shown in green in Figure 2B, with volume 27,079,000 Å^3^ and surface area 1,487,713 Å^2^. This void encapsulates the capsid and the genomic nucleic acid of the virus. The corresponding sphericity index (computed as the ratio of the surface area of a sphere with the same volume as the void to the surface area of the void) is 0.3, i.e., relatively low. This comes from the fact that the surface is not smooth, as it adapts to the local geometry of the residues that face the inside of the outer shell.

#### 2.3.2. The E Protein–M Protein Complex

**Figure 3:**
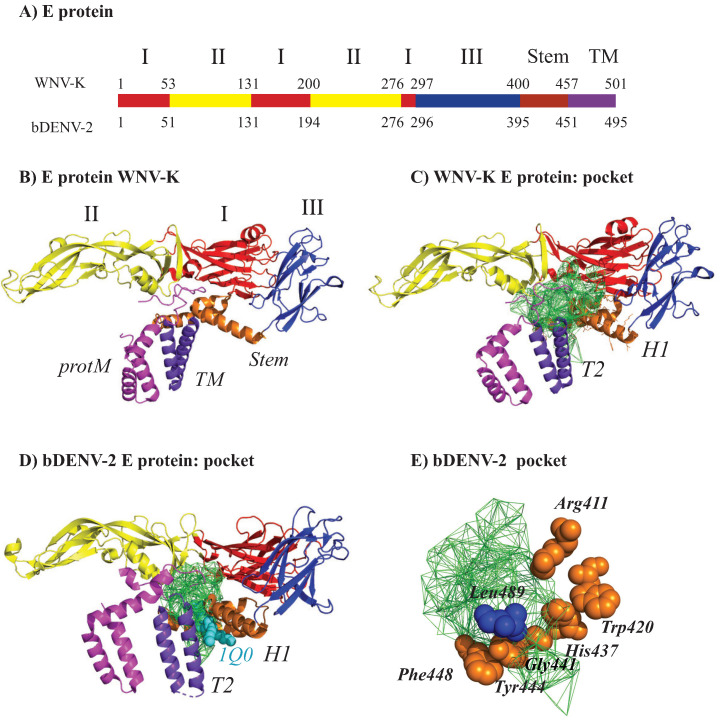
The geometries of the E protein–M protein complex of WNV-K virus (PDB code 7KVA) and of a chimeric bDENV-2 virus (PDB code 7KV8). (**A**) The E proteins of the two viruses have similar architectures (see text for details). They include five domains, i.e., I (red), II (yellow), III (blue), the stem (orange), and the trans membrane domain, TM (purple). (**B**) Cartoon representation of the E protein of WNV-K, using the same color scheme as in panel A. (**C**) The main pocket identified by UnionBall in the E protein—M protein complex of WNV-K is shown in green. (**D**) The same pocket, but for the chimeric bDENV-2 virus. Note that the two pockets for WNV-K and bDENV-2 are at a similar location; those locations map with the lipid–ligand pocket identified by Hardy et al. [110]. The lipid ligand 1Q0 observed in the structure for bDENV-2 E protein fits within this pocket. (**E**) The positions of the contact residues between the E protein of bDENV-2 and the lipid ligand pocket [110] in relation with the pocket identified by UnionBall. Panels (**B**–**D**) were generated using Pymol [111].

The E protein monomer of *flaviviridae* viruses comprises three domains, I, II, III, and a transmembrane segment, itself composed of a stem and a transmembrane element, TM. Domain I serves as a scaffold to the global organization of the E protein structure, domain II includes the dimerization interface and two glycosylation sites, and the peptide of fusion with the cellular membrane, while domain III is compact and immunoglobulin-like [112,113]. E proteins form dimers on the outer shell of mature *flaviviridae* viruses. The protein M is located underneath the E protein. We repeated the analysis described above on a single copy of the E protein–M protein complex from two viruses, the WNV Kunjin viruses already analyzed above (PDB code 7KVA), and of the chimeric Binjari virus-Dengue virus type 2 (bDENV2). The genome of this chimera includes the nonstructural genes from the Binjari virus and the structural genes of the WNV Kunjin virus [110]. Figure 3A shows the dual complex of the union of balls representing the E protein–M protein complex for WNV; the dual complex for the bDENV2 complex is very similar (not shown). In Figure 3B,C, we show the main pocket identified by UnionBall within WNV and bDENV2, respectively. In both cases, the pocket is found around the H1, H2, H3 helices from the stem region and the T2 helix from the TM region. Hardy et al. identified a lipid-binding site at the same position in bDENV2 and showed that this pocket is conserved among vertebrate-infecting flaviviruses [110]. Note that the lipid ligand 1Q0 that they used in their experiments, present in the structure of bDENV2 (7KV8), fits within the pocket detected by UnionBall. Hardy et al. also identified residues Arg411, Trp420, His437, Gly441, Tyr444, Phe448, and Leu489 at the surface of, and essential for, the lipid binding pocket. In Figure 3C, we show that those residues are indeed at the surface of the pocket identified by UnionBall.

### 2.4. Application of UnionBall to Modeling: A Toy Problem on the Capsid Protein of Flaviviruses

As mentioned above, detecting and measuring internal cavities of biomolecules is often performed as a first step for drug design. Here, we demonstrate how UnionBall can be used to perform such as task on a protein structure available in the PDB dataset and how this information can be used for a protein whose structure is not yet known.

The genome of a flavivirus encodes for a large polyprotein. This protein is cleaved by host and viral proteases to generate three structural proteins, the C or core protein, the prM or membrane protein, and the E or envelope protein, as well as several nonstructural (NS) proteins. The geometries of the E protein and prM protein were described above. Here, we are concerned with the C protein that is used by the virus to form a spherical or isometric nucleocapsid or core to encapsulate its genome. High-resolution structures of the C protein are available for WNV [114], Zika [115], and DENV serotype 2 [116] obtained by X-ray crystallography. The C protein usually forms dimers that are then organized in tetramers which form long filamentous ribbons in the crystal. The quaternary organization of the C protein plays a central role in the whole virus maturation process [117], and as such, has been a target for drug design against flaviviruses. For example, ST148 is an inhibitor of Dengue virus that targets the capsid protein [118]. It was shown to induce a “kissing” interaction between two protein C dimers, resulting in virions that are then defective when the new virus infects new cells (i.e., the nucleocapsid does not separate to release the viral genome) [116]. Understanding how ST148 binds to the C protein is, therefore, important for understanding its inhibition properties.

**Figure 4:**
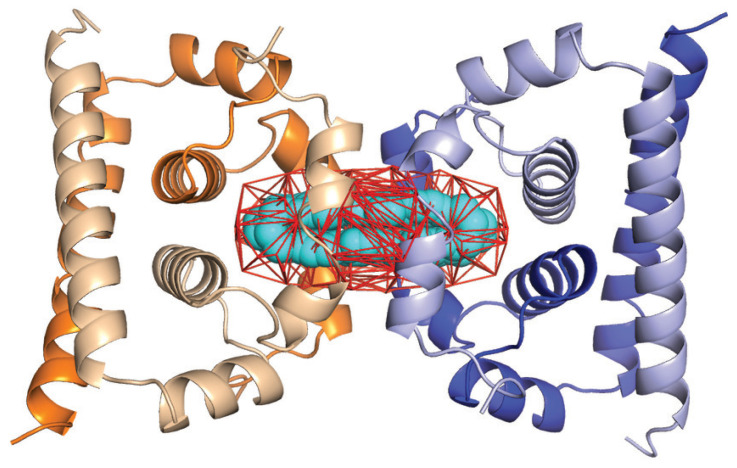
The largest pocket (red) superimposed onto the structure for DENV-2 C protein tetramer (PDB code 6VG5), with the four monomers show in cartoon mode in light blue, blue, wheat, and orange. The inhibitor ST148 is shown using a ball model in cyan. The pocket is computed with UnionBall, with a water probe of 2.0 Å. The figure was generated using Pymol [111].

We analyzed the geometry of the C protein tetramer of DENV-2 using UnionBall. The PDB structure 6VG5 [116] corresponds to this protein bound to the inhibitor ST148. All ligands, ions, and crystallographic water molecules were removed prior to running UnionBall. UnionBall only includes one parameter, i.e., the radius of the water probe. We set it to 2.0 Å, to detect deep pockets within the tetramer. In Figure 4, we show the main pocket detecting by UnionBall superimposed on the PDB structure of the C protein tetramer in the presence of the inhibitor. As observed, the inhibitor fits exactly within the pocket that sits at the interface between two protein C dimers, forming a tight lock between those dimers.

**Figure 5:**
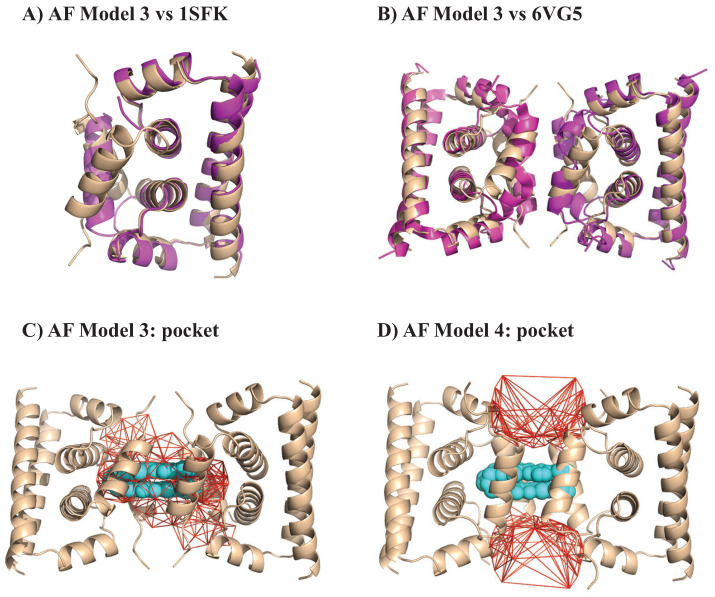
Modeling the interaction of the inhibitor ST148 [118] with a tetramer of C protein from WNV. (**A**) Superposition of the experimental structure of a C protein from WNV (PDB code 1SFK, [114]), in magenta, with the third model generated by AlphaFold2 using ColabFold [119], in wheat color. The third model is the closest to the experimental structure, with an RMS of 0.71 Å. (**B**) Superposition of the AlphaFold2 tetramer model 3 of the C protein from WNV (see text for detail), in wheat, with the experimental structure of the equivalent tetramer of DENV-2 [116], in magenta. The overall RMS is 1.2 Å. (**C**) The largest pocket (red) superimposed onto model 3 for the WNV tetramer of C protein. The putative position of the inhibitor ST148 is shown using a ball model in cyan. (**D**) The two largest pockets (red) superimposed onto model 4 for the WNV tetramer of C protein. The inhibitor ST148 is shown in cyan. The figure was generated using Pymol [111].

We then assessed if the same inhibitor would exhibit the same fit for a C protein tetramer of WNV. A structure exits for such a tetramer, under the PDB code 1SFK [114]; however, it is missing a large portion of the N-terminal region of two of its monomers, a region that is unfortunately part of the interface between two dimers where the inhibitor binds. To circumvent this problem, we used ColabFold, the open interface to AlphaFold2 [119], to generate five models of the full structure of a C protein dimer. We compared all five models to the conformation of the incomplete C protein dimer in 1SFK: all those models show remarkable resemblance to the experimental structure, with RMS deviations in the range 0.7 Å to 0.9 Å over 717 atoms (RMS calculations were performed using the “align” function of Pymol [111]). In Figure 5A, the superposition of model 3 with the experimental structure 1SFK is shown. We then align the five different model dimers from AlphaFold with both dimers of the experimental structure of DENV-2 C protein tetramer [116], using the function align from Pymol. Again, the resulting models resemble remarkably the experimental structures with RMS varying from 1.1 to 1.2 Å; this is illustrated for model 3 in Figure 5. We then analyzed the geometry of those model tetramers using UnionBall, each time setting the radius of the probe to 2 Å. We observed two different behaviors over the five models, which are illustrated in Figure 5. Models 1 and 3 exhibit a central pocket at the interface between the two protein C dimers which matches the position of the ST148 inhibitor, see Figure 5C. This pocket, however, is larger than the volume of the inhibitor, and larger than the pocket observed for the DENV-2 tetramer (see Figure 4). In contrast, models 2, 4, and 5 do not exhibit a similar pocket; they include instead two large pockets that would outflank the putative position of the inhibitor, as observed in Figure 5D.

The modeling proposed above would indicate that if ST148 binds to the C protein of WNV, this binding would not be as strong as its binding to the C protein of DENV-2. It is known that ST148 shows efficacy only to serotype 2 of DENV [116]; to our knowledge, its effect on WNV is not known. Our results, however, should be considered with caution. We have used structural models generated by AlphaFold to analyze the geometric interactions between the C protein of WNV with ST148; while these models seem remarkably accurate, we already observe two different behaviors that are probably associated with different positions of sidechains at the interface between two dimers. AlphaFold2 is known to yield mixed results for drug design [45]. Ultimately, our results should be validated experimentally.

## 3. Dynamics of Viral Structures

### 3.1. Motivation

In the previous section, we considered the fixed geometry of biological structures. Geometry is, however, one facet of the problem of characterizing such molecules. Indeed, biological function arises from action, i.e., the dynamics of molecule. The standard approach to simulating such dynamics is to solve numerically the Newton equations associated with all its atoms. Newton equations are second-order partial differential Equations (PDE). They are usually solved incrementally as a function of time. The corresponding step size in time is extremely small (in the order of a femtosecond) if we want accurate solutions. This leads to the need to compute the energy of the molecular system under study a large number of times. One evaluation of the energy is of order O(NlogN), with *N* being the total number of atoms in the system. For large values of *N*, say in the millions, such a calculation, and more importantly its repeats, become computationally prohibitive. Many efforts are underway to either design specific hardware to solve those PDEs or to improve the algorithms to compute the energy values [120,121,122,123,124,125,126]. These efforts allow for molecular dynamics simulation of systems with up to 100 million atoms [60,127,128,129,130]. It should be noted, however, that those successes still rely on the availability of specific hardware such as ANTON [120] or of a large supercomputer.

An alternate and promising approach to standard molecular dynamics is to infer dynamics directly from static structures corresponding to locally stable states [131], together with reliable coarse-graining approaches to bridge the time-scale gap [132,133]. Cartesian Normal Modes, for example, represent a class of movements around a local energy minimum that are both straightforward to calculate and have been found to be biologically relevant [134,135,136]. Normal mode techniques have been used extensively to study the dynamics of virus structures (for reviews, see for example Refs. [137,138,139]). In the following, we present one such technique. A more detailed presentation is available in the original papers [140,141].

### 3.2. Methodology

Computing coarse-grained normal modes for a molecular system is deceptively simple. We start with a conformation $X_{0}$ for the molecular system and a coarse-grained potential *V* that is minimum at $X_{0}$. We take a second-order approximation of that potential:

$$\begin{array}{c} V\left(X\right)\approx V\left(X_{0}\right)+\nabla V{\left(X_{0}\right)}^{T}\left(X-X_{0}\right)+\frac{1}{2}{\left(X-X_{0}\right)}^{T}H\left(X_{0}\right)\left(X-X_{0}\right), \end{array}$$

where $\nabla V(X_{0})$ and $H(X_{0})$ are the gradient of the potential and the Hessian of the potential, both at $X_{0}$, respectively. Since *V* is minimum at $X_{0}$, $\nabla V(X_{0})=0$, and as $V(X_{0})$ is a constant, it will not influence the dynamics of the system. We then define a normal mode potential $V_{NM}$ at a position X in the neighborhood of $X_{0}$ as follows:

$$\begin{array}{ccc} V_{NM}\left(X\right) & = & V\left(X\right)-V\left(X_{0}\right)\\ & = & \frac{1}{2}{\left(X-X_{0}\right)}^{T}H\left(X_{0}\right)\left(X-X_{0}\right). \end{array}$$

The equations of motion defined by the potential $V_{NM}$ are derived from Newton’s equation:

(3) $$\begin{array}{ccc} \frac{d^{2}X}{dt^{2}} & = & -\nabla V_{NM}\left(X\right)\\ & = & -H\left(X_{0}\right)\left(X-X_{0}\right). \end{array}$$

The “normal modes” of the system are oscillatory motions of the system (also called concerted motions). The trajectory of the system under a normal mode *k* then has the following form:

(4) $$\begin{array}{c} X_{k}=A_{k}\alpha_{k}\cos\left(\omega_{k}t+\delta_{k}\right), \end{array}$$

where $A_{k}$ is a vector that defines which atoms are involved in this specific normal mode, $\alpha_{k}$ is the amplitude, $\omega_{k}$ is the frequency of the mode (i.e., how fast is oscillates), and $\delta_{k}$ is a phase shift. The normal mode is a solution of the Newton equations. Replacing Equation (4) in Equation (3), we find that the normal modes are characterized with the following eigenvalue problem:

(5) $$\begin{array}{c} HU=U\Omega . \end{array}$$

The frequencies of the modes $\omega_{k}$ are given by the elements of the diagonal matrix Ω, namely $\omega_{k}^{2}=\Omega \left(k,k\right)$. The vectors $A_{k}$ are the eigenvectors and correspond to the columns of the matrix *U*, and the amplitudes and phases, $\alpha_{k}$ and $\delta_{k}$, are determined by initial conditions. We note that the first six eigenvalues in Ω are equal to 0, as they correspond to global translations and rotations of the biomolecule. Note that for simplicity, we assumed that each atom is assigned a mass of 1. The procedure described above can easily be expanded to the more general case of different values for the atomic masses.

#### 3.2.1. Coarse Grained Potentials for Normal Mode Analysis of Biomolecules

A typical semi-empirical potential function used in classical molecular simulation has the following form [142,143,144,145,146]:

$$\begin{array}{ccc} U & = & \sum_{b}k_{b}{\left(r_{b}-r_{b}^{0}\right)}^{2}+\sum_{b}k_{a}{\left(\theta_{a}-\theta_{a}^{0}\right)}^{2}\\ & & +\;\sum_{t}k_{t}\left(1+\cos n(\phi_{t}-\phi_{t}^{0})\right)\\ & & +\;\sum_{i<j}\left(\frac{A_{ij}}{r_{ij}^{12}}-\frac{B_{ij}}{r_{ij}^{6}}+\frac{q_{i}q_{j}}{Dr_{ij}}\right). \end{array}$$

where the terms in the first three sums represent bonded interactions: covalent bonds, valence angles, and torsions around bonds. The two terms in the last sum represent nonbonded interactions: a Lennard-Jones potential for the van der Waals force and the Coulomb potential for electrostatics. This sum usually excludes pairs of atoms separated by one, or two covalent bonds. The force constants, *k*, the minima, $r^{0}$, $\theta^{0}$, and $\phi^{0}$, the Lennard Jones parameters, *A* and *B*, and the atomic charges *q* define the force field. They are derived from data on small organic molecules, from both experiments and ab initio quantum calculations.

Such potentials were used for normal mode analyses from their inception [134,135,136]. There are, however, drawbacks. The method presented above assume that the initial conformation $X_{0}$ is a minimum of the potential. Finding such a minimum for the potential described above is difficult for a large system. If this minimum is not exact, the Hessian at the minimum may have negative eigenvalues that are not physical. The Elastic Network Model (ENM) was originally introduced by [147] to circumvent this problem. It is a model that captures the geometry of the molecule of interest in the form of a network of inter-atomic connections, linked together with elastic springs. Its potential is a quadratic energy on the inter-atomic distances, defined as follows:

(6) $$\begin{array}{c} V_{T}\left(X\right)=\frac{1}{2}\sum_{\left(i,j\right)}k_{ij}{\left(r_{ij}-r_{ij}^{0}\right)}^{2} \end{array}$$

when the biomolecule is in conformation X. The $k_{ij}$ are the force constants of the “spring” formed by the pairs of atoms *i* and *j*, $r_{ij}$ and $r_{ij}^{0}$ are the distances between atoms *i* and *j* in the conformation X and $X_{0}$, respectively. In the initial formulation proposed by Tirion [147], the sum includes all pairs of atoms (i,j) that satisfies $r_{ij}^{0}<R_{c}$, where $R_{c}$ is a cutoff distance. Note that $V_{T}\left(X_{0}\right)=0$ and $\nabla V_{T}\left(X_{0}\right)=0$ by construction.

The potential given by Equation (6) is a simple pairwise geometric potential. As such, it does not account for the stereochemistry of the molecule explicitly. In particular, it may not preserve bond lengths, bond angles, and preferences in dihedral angles. A possibly better potential would include those bonded interactions explicitly. Such a potential was originally proposed by Nobuhiro Gō [148] and later adapted to the framework of coarse-grained normal modes [149,150]. It only considers the Cα of all residues in the molecule of interest. If we define as $b_{i}$ the length of the pseudo-bond between the Cαs of the consecutive residues *i* and i+1, $\theta_{i}$ the virtual bond angle formed by the Cαs of the consecutive residues *i*, i+1, and i+2, and $\phi_{i}$ the virtual dihedral angle formed by the Cαs of the consecutive residues *i*, i+1, i+2, and i+3, the Go potential at a conformation X is defined as:

(7) $$\begin{array}{ccc} V_{G}\left(X\right) & = & V_{bond}\left(X\right)+V_{angle}\left(X\right)+V_{dih}\left(X\right)+V_{nb}\left(X\right)\\ & = & \frac{1}{2}\sum_{i=1}^{N-1}K_{r}{\left(b_{i}-b_{i}^{0}\right)}^{2}+\frac{1}{2}\sum_{i=1}^{N-2}K_{\theta }{\left(\theta_{i}-\theta_{i}^{0}\right)}^{2}\\ & & +\sum_{i=1}^{N-3}\left[K_{\phi 1}\left(1-\cos\left(\phi_{i}-\phi_{i}^{0}\right)\right)+K_{\phi 3}\left(1-\cos3\left(\phi_{i}-\phi_{i}^{0}\right)\right)\right]\\ & & +\sum_{\left(i<j-3\right)}\epsilon \left[5{\left(\frac{r_{ij}^{0}}{r_{ij}}\right)}^{12}-6{\left(\frac{r_{ij}^{0}}{r_{ij}}\right)}^{10}\right], \end{array}$$

where the superscript 0 refers to the values of the variables for the conformation $X_{0}$. The first three terms refer to (pseudo-) bonded interactions, while the last term corresponds to nonbonded interactions. Note that the molecular system considered includes multiple chains (such as a virus outer shell), special care is needed to only include bonds, angles, and dihedral angles that exist within a chain.

As written in Equation (7), the nonbonded term in the Go potential is written as a sum over all pairs of Cα atoms that are more than three indices away along the sequence. In practice, however, only a subset of those pairs are considered. This subset can be built using a cutoff $R_{c}$, as described for the Tirion potential. An alternative is to include all pairs that form an edge in the Delaunay triangulation of the Cα atoms of the molecular system. Using the Delaunay triangulation has two advantages: it reduces the number of pairs i,j considered and it alleviates the need to set a value for $R_{c}$. It was shown that filtering the pair of atoms based on the Delaunay defines normal modes that reproduce protein dynamics at least as well as a filtering based on a cutoff distance [151].

#### 3.2.2. Diagonalizing the Hessian Matrix

The core of a normal mode analysis is the computation of the eigenvalues and eigenvectors of the Hessian of the potential, as described in Equation (5). While solving this task is standard in linear algebra and many packages have optimized routines for eigenanalysis, such as LAPACK [152], there are two main issues to consider when trying to use them for a very large molecular system. First, there are storage issues. The full Hessian matrix requires storage of the order $O(N^{2})$, where *N* is the number of atoms included in the calculation. Such a level of storage can be prohibitive when *N* is of the order of tens of thousands. Second, standard algorithms for computing eigenpairs of a matrix are of order $O(N^{3})$ in computing complexity, again prohibitive for large systems. There are, however, solutions to both problems that we briefly describe here.

(i) *The storage issue.* As described above, the pairs of atoms that are included in the potential are filtered based on either a cutoff value or based on a geometric construction such as the Delaunay triangulation. As a consequence, the Hessian matrix is sparse, with the number of nonzero values only a fraction of the expected $O(N^{2})$, and more of the order O(N) (see for example [151]). In addition, the forms of both the Tirion potential and the Go potential are such that their Hessian can be expressed as sums of tensor products, further reducing their storage needs [153].
(ii) *Computing eigenvalues and eigenvectors*. In her original paper on coarse-grained normal mode analyses of proteins, Tirion showed that the lowest frequency normal modes based on a geometric potential capture most of the dynamics of the molecular system of interest [147]. She did not indicate, however, how many low frequency normal modes need to be considered, as this is most likely problem specific (see for example [154]). Still, only a fraction of the total eigenvalues and eigenvectors of the Hessian matrix need to be computed [131]. There are powerful iterative algorithms for computing a subset of the eigenpairs of a matrix. In Ref. [141], we compared four such methods, namely an implicitly restarted Arnoldi method as implemented in ARPACK [155], a simple modification of this method based on polynomial filtering [156,157], a variational method based on the minimization of an energy function [138,158], and a block Chebyshev–Davidson method [159,160]. We have shown that the latter provides the most efficient implementation when computing eigenpairs of extremely large Hessian matrices corresponding to large viral structures [141].

#### 3.2.3. Correlated Motions within a Molecular System

The Boltzmann distribution for the approximate potential of quadratic potential used for coarse-grained normal mode analyses corresponds to a multivariate Gaussian distribution whose covariance matrix is proportional the inverse of the Hessian *H*. As the six lowest normal modes have frequencies equal to 0 (they correspond to the three degrees of freedom associated with translations and the three degrees of freedom associated with rotations), the inverse of *H* is not defined. It is possible to compute a pseudo-inverse that corresponds to the covariance matrix of internal deformation:

$$\begin{array}{c} C=\sum_{k=7}^{M}\frac{1}{\omega_{k}^{2}}A_{k}A_{k}^{T} \end{array}$$

where $\omega_{k}$ and $A_{k}$ are the k−th eigenvalues and eigenvectors, respectively. The summation starts at k=7, i.e., at the first nonzero mode, and stops at a prescribed *M*, i.e., the highest frequency mode considered. The correlation of the motions of two atoms *i* and *j* is then defined as [161]:

$$\begin{array}{c} P_{ij}=\frac{tr(C_{ij})}{\sqrt{tr\left(C_{ii}\right)tr\left(C_{jj}\right)}} \end{array}$$

The values $P_{ij}$ range from −1 to +1. They are stored in a matrix which we refer to as the Cross Correlation Matrix (CCM).

### 3.3. Examples

We have implemented the algorithm described above in the program NormalModes [141,153]. We used it to analyze the dynamics of the outer shell of WNV-K, whose geometry is studied above. We use the Go potential to represent the energy of the molecular system. As the Go potential is computed from the Cα atoms, we isolated those from the PDB file 7KVA. We excluded the M protein and only considered the E protein. The Go potential was parametrized as follows: $K_{r}=100\epsilon$, $K_{\theta }=20\epsilon$, $K_{\phi 1}=\epsilon$, $K_{\phi 3}=0.5\epsilon$, with ϵ=0.36. In addition, the nonbonded term $V_{nb}$ is computed over all edges of the Delaunay complex associated with the Cα atoms of the outer shell. We refer to the union of those edges as the Elastic Network (EN) of the virus outer shell.

We considered the E protein in four different environments: isolated, MONO, corresponding to chain A in the asymmetric unit of 7KVA, as a dimer, DIMER, corresponding to chain A in the asymmetric unit of 7KVA, within a raft, RAFT, and within the whole outer shell structure, FULL. The E protein forms dimers on the outer shell of the mature form of the virus. These dimers organize in the form of rafts, namely three dimers lying parallel to each other (see below). The corresponding complexes, MONO, DIMER, RAFT, and FULL contain 501, 1002, 3006, and 90,180 residues, respectively. We computed the hundred lowest normal modes for each of these complexes, using the procedure detailed above. The normal modes were computed using the empty protein shells, in accordance with previous studies of viral particles using normal mode analyses [162,163,164,165,166,167]).

#### 3.3.1. Characterizing the Low-Frequency Normal Modes of WNV-K

**Figure 6:**
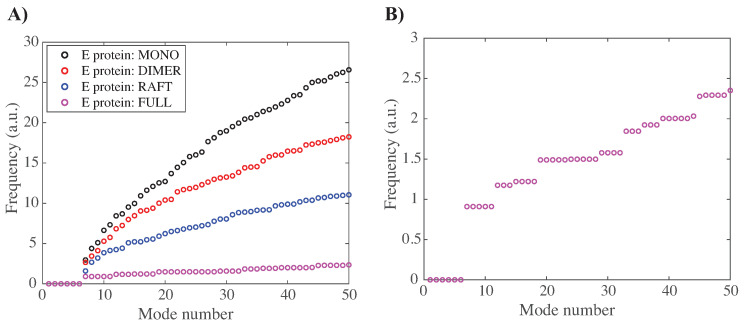
Comparing the low frequencies of the normal modes of the E protein of WNV-K in different environments. (**A**) The frequencies of the first fifty normal modes of the MONO (black circles), DIMER (red circles), RAFT (blue circles), and FULL (magenta circles) complexes of WNV-K (see text for details on the complexes). Note that those frequencies are in arbitrary units, as the parameters of the Go potential are also in arbitrary units. The amplitudes of those frequencies decrease as the size of the complex increases. (**B**) The frequencies of the first 50 normal modes for the full outer shell, FULL; note the degeneracies of the normal modes, which are a consequence of the symmetries in an icosahedral geometry.

In Figure 6A, we compare the frequencies of the first fifty normal modes of the MONO, DI, RAFT, and FULL complexes of WNV-K. The first six frequencies are found equal to zero, for all complexes considered. This is expected, as those frequencies correspond to the rigid motions (three translations and three rotations) that do not affect the Go potential. Indeed, all terms in the potential can be expressed with interatomic distances only, and therefore, are conserved under translations and rotations of the molecular system. The larger the protein complex, the more the spectrum of frequencies of its normal modes move to lower frequencies. This is indicative of the presence of more collective motions in protein oligomers. Figure 6B, which plots the lowest frequencies for the full outer shell of WNV-K, reveals the presence of degeneracy, namely repeating frequencies. These repeats are a consequence of symmetries in the outer shell, as it is icosahedral.

#### 3.3.2. Concerted Motions of E Proteins in Different Environments

**Figure 7:**
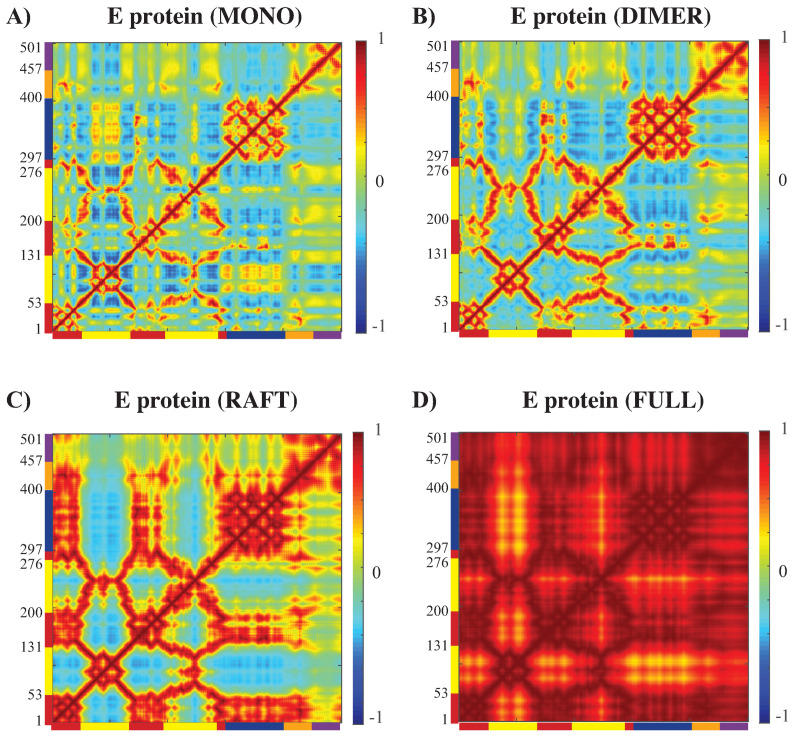
Correlated motions in the E protein from the WNV-K outer shell. Cross Correlation Matrices (CCM) obtained from the 94 first nonzero modes for the E protein alone (MONO, (**A**)), the E protein in a dimer (DIMER, (**B**)), the E protein in a raft (RAFT, (**C**)), and the E protein in the whole outer shell of WNV-K (FULL, (**D**)). Those plot show correlations between the motions of Cα atoms in each complex considered. Both axes represent the amino acid residue indices. Each pixel in the image corresponds to an element of the CCM matrix. It shows the correlation between the motions of Cα atoms from two residues in the protein in a range from −1 (anticorrelated, blue) to 1 (correlated, red), with 0 denoting the absence of correlation. The color code for the X and Y axes of the CCM plots in (**A**–**D**) follows the standard designation of the E protein domains, I (red), II (yellow), III (blue), stem (orange), and transmembrane domain (purple) (see caption of Figure 3 for details).

The cross correlation matrices (CCM, see methodology above) for the E protein vary significantly between the MONO, DIMER, RAFT, and FULL complexes, as observed in Figure 7. The CCM for the E protein alone reveals significant positive correlations within each of the three domains, i.e., I, II, and III. Inter-domain residue pairs from domains II and III show both positive and negative correlations in their atomic fluctuations, while residues in domain I are only weakly correlated with residues of domain II and III. When the dynamics of the E protein are studied in the context of the asymmetric unit, the same positive correlations are observed within each of the three domains. The interactions between the domains change significantly, however, as we consider the E protein in multimeric structures. In the RAFT complex, residues in domain II are strongly anticorrelated with residues from domain III, while residues in domain I are strongly positively correlated with residues in domain III. The DIMER complex shows behavior that are between those of the MONO and RAFT complex. The full outer shell shows globally positive correlations for all pairs of residues within the E protein; those correlations are the result of concerted dynamics within the outer shell. In the MONO and DIMER complexes, the stem and transmembrane domains show weak negative correlations with domain II. These correlations become positive in the RAFT and FULL complexes.

#### 3.3.3. Concerted Motions of Rafts of E Proteins in Different Environments

**Figure 8:**
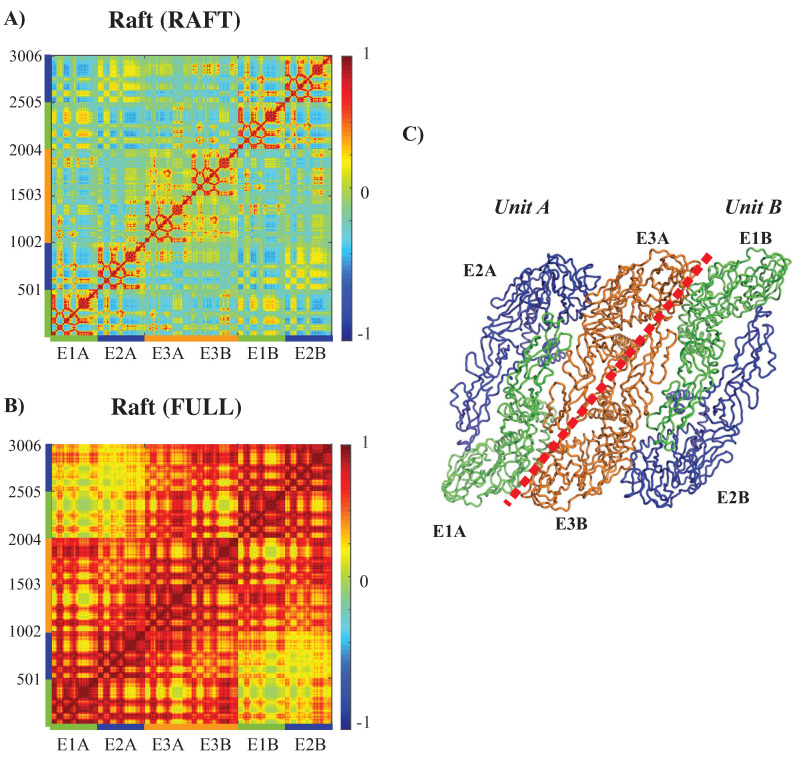
Correlated motions in an E protein raft. Cross Correlation Matrices (CCM) obtained from the 94 first nonzero modes for an E protein raft alone (RAFT), and a raft in the whole outer shell (FULL) for WNV-K (panels **A**,**B**). X axes and Y axes are residue indices. The positions of the six E proteins of the raft are indicated, with labels and color codes defined on the structure in (panel **C**). (**C**) Cartoon model for the raft. Note that a raft includes two asymmetric units, labeled Unit A and Unit B. The third E protein of each unit, E3A and E3B, form a dimer. Panel E was generated using Pymol [111].

Figure 7 reveals the effects of packing in the viral outer shell on the dynamics of one E protein. We performed a similar analysis on a larger structure of the outer shell, namely a raft. A raft is formed from six E proteins organized as three dimers arranged in a parallel manner (see Figure 8C). The whole outer shell contains 30 such rafts. In Figure 8A,B, we analyze the extent to which packing influences the dynamics of such rafts for WNV-K.

The CCM matrix of the raft by itself (i.e., defined by the complex RAFT) clearly identifies the six E proteins along the diagonal, as observed on Figure 8A. Each of those E proteins exhibits dynamics correlation patterns equivalent to those observed in the E protein when it is in the RAFT complex. The interactions between the E proteins are consistent with the structure of the raft. The E proteins E3A and E3B, which form a dimer between two asymmetric units, show strong positively correlated dynamics. In contrast, proteins E1A and E2A in Unit A, and proteins E1B and E2B in Unit B have a pattern of interactions that include both positively correlated and negatively correlated motions, depending on their domains: for example, domains III have negative correlations between the two proteins, while domains II are positively correlated between the two proteins. The pair of proteins (E1A, E2A) shows weak correlated dynamics with the pair of proteins (E1B, E2B), with a chessboard pattern (i.e., positive correlations between E1A and E1B, and negative correlations between E1A and E2B).

The CCM for a raft included in the whole outer shell (Figure 8B) reveals different patterns than those described for the raft alone, highlighting again the impact of packing in the whole virus environment. There is a high level of positive correlation of motions within each of the units A and B. The proteins E3A and E3B that form a dimer at the center of the raft are interacting with themselves in the raft alone, while they show strong levels of positive correlations with all the E proteins of the same asymmetric unit in the raft when considered within the whole outer shell. Such a behavior would favor concentration of concerted internal motions within a few E protein dimers at the center of the rafts in the whole outer shell instead of a more uniform dissemination of concerted motions.

Similar behaviors have been observed for the outer shells of Dengue virus and ZIKA virus [140,153].

#### 3.3.4. Computing Time for NormalModes

**Figure 9:**
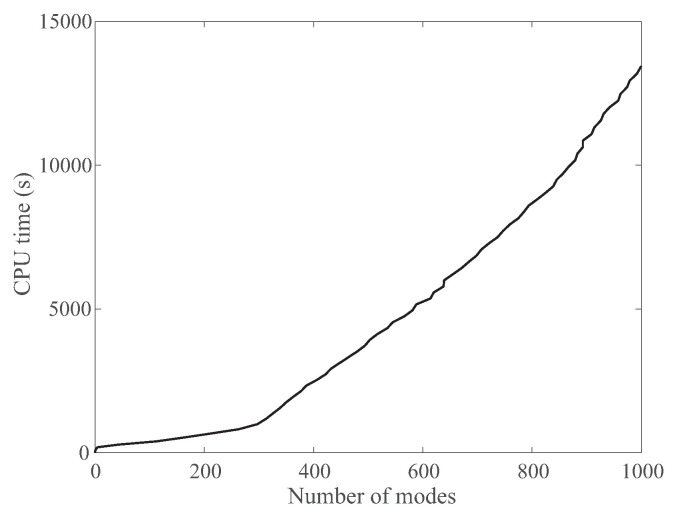
Total CPU time (wall time) for NormalModes as a function of the number of converged eigenvalues of the Hessian for all E proteins of the outer shell of WNV-K. Only Cα atoms are considered as we consider the Go potential. There are 90,180 Cα atoms within the whole outer shell (with only E proteins considered). The Delaunay complex associated with those atoms that form the list of nonbonded interactions includes 503,287 edges. The computation is performed on a quad-core Intel Core I7 processor running at 4.0 GHz with 64 GB of RAM.

The procedure described above and implemented in the program NormalModes was developed to enable the analysis of the dynamics of large molecular systems, including viruses on standard desktop computers. To ascertain that this is indeed the case for NormalModes, we measured the computing required to evaluate up to 2000 normal modes for the whole outer shell of WNV-K. As described above, this outer shell is large, including more than 90,000 residues. Using the Go potential, this means that we need to perform a normal mode calculation over 90,000 atoms. In theory, the corresponding Hessian for the Go potential is a matrix of size 270,000 × 270,000, whose storage would require more than 580 GB of memory, a number that is not compatible with a desktop computer. We have shown, however, that there are ways to circumvent this issue. We show the actual cumulative CPU wall time needed to compute the first 1000 normal modes associated with this matrix as a function of the number of modes computed in Figure 9. The total computing time is found to be approximately piece-wise linear, with a change of slope at around 300 modes corresponding to a slow down after those 300 modes. Computing those first 300 modes is relatively fast (around 1000 s, i.e., 17 min), considering that the CPU we used, a quad-core Intel Core I7 processor running at 4.0 GHz, is not state-of-the-art. Such a computing time is often deemed acceptable; the results presented above also highlight that 300 modes may be enough to analyze the dynamics of WNV-K. In comparison, computing 1000 modes requires 13,600 s on the same processor, i.e., approximately 3 h 45 min, while computing 4000 modes requires 130,000 s, i.e., 36 h.

## 4. Energetics of Viral Structures

### 4.1. Motivation

Vaccine and antiviral drugs are considered to be the most effective options for the prevention and treatment of virus-induced diseases. Vaccines, for example, have proven to be effective to curb the impact of the SARS-CoV-2 pandemics, reducing both mortality and morbidity associated with its associated disease, COVID-19, by limiting the number of infections (see for example Ref. [168]). However, a vaccine is only effective if it matches the prevalent viral strain. For example, it is a challenge each year to assess the composition of the current influenza vaccine early enough to allow time for the manufacture and distribution of the most up-to-date vaccine. In parallel, drugs that are designed to inhibit specific targets of viruses often suffer from the same problem. They are, however, the solutions for treating the associated diseases when infection has occurred.

Drugs are natural or nonnatural ligands that are developed to inhibit the function of molecules, usually proteins, that are essential for the virus life cycle. The inhibition is the result of a tight binding of the ligand on the target protein. The identification and characterization of such binding sites are, therefore, essential steps in structure-based drug design (for reviews, see [169,170,171]). Many of the corresponding methods are fast and easy to implement, but rely on severe simplifications. Geometric methods, for example, assume a static structure for the target protein. They identify pockets within the protein and do not consider the nature of the residues bordering those pockets, and even the geometry of the putative ligand. This is true for our own method, UnionBall, presented above, and for other similar techniques [169,172]. In contrast, energy-based methods combine geometry to position a putative ligand near the protein of interest with energy calculation to estimate the likeliness of their interactions. Q-SiteFinder [172], for example, coats the protein surface with a layer of methyl probes and calculate van der Waals interaction energies between the protein and those probes. Probes with favorable interaction energies are retained and clusters of these probes are deemed to define putative binding sites. Other methods follow the concept of fragment-based drug discovery (FBDD) in which first small chemical fragments are identified as possible binder to the target, and then combined to produce a ligand with a high affinity [173,174]. These methods include GRID [175], MCSS [176], and FTMAP [177,178]. These techniques, however, often yield a large number of false-positive energy minima.

Sampling of the conformation of the ligand, the protein, and the environment within the putative binding site, including the presence of ordered water molecules and salt, is necessary for a computational technique to successfully identify drug-binding sites. Molecular dynamics (MD) simulations provides a framework for such sampling [179,180,181,182]. When the actual ligand is not known, it is possible to incorporate co-solvents in the simulations to mimic this ligand and consequently improve the identification of binding sites [109,183,184,185,186,187,188]. As mentioned in the previous section, molecular dynamics, however, are time-consuming.

We have recently proposed an alternate approach to the grid-based drug mapping procedure and to the cosolvent-enhanced MD simulations described above. This approach, with the acronym HDPBL (see below) is based on multi-probe exploration. It generates the densities of dipoles representing a polar solvent, of anions, cations, as well as the densities of hydrophobic cosolvent molecules, thereby enabling the identification of polar, positively charged, negatively charged, and hydrophobic binding sites on the target protein simultaneously. In the following, we briefly present HDPBL. A more rigorous presentation is available in the original paper [189].

### 4.2. Methodology

#### 4.2.1. A Lattice Gas Model for the Environment of the Solute of Interest

**Figure 10:**
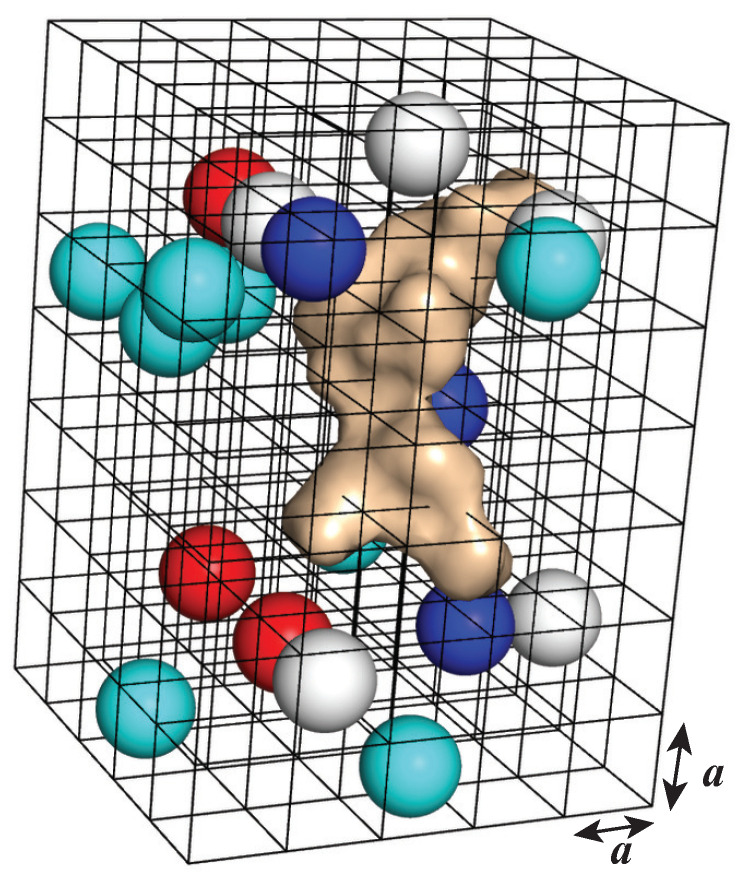
Schematic illustration of the lattice gas model. Each lattice cell may be empty, occupied by one ion (red for positive ions, blue for negative ions), a water molecule (cyan), or an inert hydrophobic particle (white). We assume here that all those species have the same size, with diameter *a*, the lattice spacing. The solute is at the center of the lattice. It is identified by its surface area (colored here in light orange (wheat)).

We use a lattice gas formalism to represent the environment around the target molecule (see Figure 10). This lattice allows us to model steric repulsion among the particles in this environment. The water molecules are distributed on this lattice. They are represented as orientable dipoles of constant module $p_{0}$. Similarly, the ions (positive and negative) are represented as spheres carrying a charge $+ze_{c}$ or $-ze_{c}$, where $e_{c}$ is the elementary electronic charge. We also include inert hydrophobic molecules. All those molecules are represented as hard spheres with equal radii a/2, where *a* is the lattice spacing. The solute itself is described by a charge density $\rho_{f}\left(r\right)$ corresponding to its fixed charges, and a hydrophobic density $\rho_{h}\left(r\right)$ corresponding to its hydrophobic sites (usually the CH_2_ and CH_3_ groups). Its surface is modeled with an indicator function γ(r) that is zero for points r inside the envelope of the solute and one otherwise. This envelope can be taken as the molecular surface or the accessible surface of the solute; we usually use the molecular surface.

#### 4.2.2. A Free Energy Model for the Solute and Lattice Gas

Let φ(r) be the electrostatic potential at position r. Each site r in the lattice surrounding the solute contains at most one particle. Let us look at the partition function by considering each type of particle.

(i) *Water molecule*: the energy of one water dipole of constant magnitude $p_{0}$ at position r is obtained as the Boltzmann-weighted average of the interaction $-{\vec{p}}_{0}\cdot \nabla \varphi \left(r\right)$ over all orientations of ${\vec{p}}_{0}$, where φ(r) is the local electric potential:
  $$\begin{array}{ccc} Z_{w}\left(r\right)=\lambda_{w}<e^{\beta p_{0}\left|\nabla \varphi \left(r\right)\right|cos\left(\theta \right)}{>}_{\theta ,\phi } & = & \lambda_{w}\frac{\sinh\left(p_{0}\beta \left|\nabla \varphi (r)\right|\right)}{p_{0}\beta \left|\nabla \varphi (r)\right|}, \end{array}$$
  where $\beta =\frac{1}{k_{B}T}$ and $\lambda_{w}$ is the fugacity of the water (see below).
(ii) *Ions*: the energy of one ion with charge $ze_{c}$ is simply $ze_{c}\varphi \left(r\right)$. We assume that there are as many positive ions and negative ions in the environment, with charges $+ze_{c}$ and $-ze_{c}$, respectively. Then:
  $$\begin{array}{c} Z_{ion}\left(r\right)=2\lambda_{ion}\cosh\left(\beta ze_{e}\varphi \left(r\right)\right), \end{array}$$
  where $\lambda_{ion}$ is the fugacity of the ions.
(iii) *Hydrophobic particles*: The hydrophobic interactions between the hydrophobic particles are defined by a Yukawa potential:
  $$w_{Y}\left(r\right)=-\frac{w_{0}}{4\pi }\frac{e^{-\kappa_{Y}r}}{r},$$
  where r=||r||, $\kappa_{Y}=1/l_{Y}$ defines the range of the hydrophobic interaction, and $w_{0}>0$ its strength. The negative sign denotes the attractive nature of the interaction. Setting ψ(r) to be the hydrophobic field associated with this potential gives the following:
  $$\begin{array}{c} Z_{h}\left(r\right)=\lambda_{h}e^{-\beta \psi (r)}, \end{array}$$
  where $\lambda_{h}$ is the fugacity of the hydrophobic particles.
(iv) *Possible empty sites*: The system may be considered as incompressible, in which case all lattice sites are occupied by one particle, or compressible, in which case a lattice site may be empty. We model this behavior by introducing a pseudo-fugacity $\lambda_{v}$ for vacancies such that $\lambda_{v}=0$ if the system in incompressible, and $\lambda_{v}=1$ otherwise. Note that this is set once at the beginning of the analysis.

The grand canonical partition function Z(r) for the lattice site at position r is then given by (enumerating the two possible occupancies: empty, or with one dipole):

(8) $$\begin{array}{ccc} Z(r) & = & \lambda_{v}+Z_{w}\left(r\right)+Z_{ion}\left(r\right)+Z_{h}\left(r\right)\\ & = & \lambda_{v}+2\lambda_{ion}\cosh\left(\beta ze_{c}\varphi \left(r\right)\right)+\lambda_{w}\frac{\sinh\left(p_{0}\beta \left|\nabla \varphi (r)\right|\right)}{p_{0}\beta \left|\nabla \varphi (r)\right|}+\lambda_{h}e^{-\beta \psi (r)} \end{array}$$

The free energy functional for the whole grid includes the electrostatic energy, the functional form for the energy of the Yukawa field, the energy of the fixed charges and hydrophobic groups of the solute, and the logarithm of the partition function Z defined in Equation (8):

(9) $$\begin{array}{ccc} F & = & -\frac{\varepsilon_{0}}{2}\int dr{\left(\nabla \varphi (r)\right)}^{2}+\frac{1}{2w_{0}}\int dr\left({\left(\nabla \psi (r)\right)}^{2}+\kappa^{2}\psi^{2}\left(r\right)\right)\\ & + & \int dr\varphi \left(r\right)\rho_{f}\left(r\right)+\int dr\psi \left(r\right)\rho_{p}\left(r\right)\\ & - & \frac{1}{\beta a^{3}}\int dr\gamma \left(r\right)\ln\left(\lambda_{v}+2\lambda_{ion}\cosh\left(\beta ze_{c}\varphi \left(r\right)\right)+\lambda_{w}\frac{\sinh\left(p_{0}\beta \left|\nabla \varphi (r)\right|\right)}{p_{0}\beta \left|\nabla \varphi (r)\right|}+\lambda_{h}e^{-\beta \psi (r)}\right) \end{array}$$

#### 4.2.3. Solving for the Electrostatic and Hydrophobic Fields

We use the Saddle-Point Approximation from statistical physics. This method, which is also called the Mean-Field Theory, consists of minimizing the free energy defined in Equation (9) with respect to the two fields φ and ψ:

(10) $$\begin{array}{ccc} -\varepsilon_{0}\nabla^{2}\varphi \left(r\right) & = & \rho_{f}\left(r\right)-\frac{2\lambda_{ion}}{a^{3}}ze_{c}\gamma \left(r\right)\frac{\sinh\left(\beta ze_{c}\varphi \left(r\right)\right)}{Z(r)}+\\ & & \;\frac{p_{0}}{a^{3}}\lambda_{w}\gamma \left(r\right)\nabla \cdot \left(\frac{\nabla \varphi (r)}{\left|\nabla \varphi (r)\right|}\frac{g\left(p_{0}\beta \left|\nabla \varphi (r)\right|\right)}{Z(r)}\right)\\ \frac{1}{w_{0}}\left(-\nabla^{2}+\kappa_{Y}^{2}\right)\psi \left(r\right) & = & -\rho_{p}\left(r\right)-\frac{\lambda_{h}}{a^{3}}\gamma \left(r\right)\frac{e^{-\beta \psi (r)}}{Z(r)}, \end{array}$$

where Z(r) is defined in Equation (8) and

$$\begin{array}{ccc} g(x) & = & \frac{\cosh x}{x}-\frac{\sinh x}{x^{2}}. \end{array}$$

Note that φ(r)→0 and $\psi \left(r\right)\to \psi_{0}$ as r→+∞, i.e., in the bulk, far from the solute.

The meanfield equations given above fully describe the system under study. The first equation is a Dipolar Poisson–Boltzmann Langevin (DPBL) Equation [190,191,192], while the second equation is a Poisson–Boltzmann-like equation involving the hydrophobic particles in the solvent and the hydrophobic charges on the solute. As a consequence, we refer to this system of equations as the Hydrophobic Dipolar Poisson–Boltzmann Langevin equations, or HDPBL equations for short [189].

All coefficients in those equations are computed either from physical constants or from input information describing the system, with the exception of the fugacities and $\psi_{0}$, which we derive now.

#### 4.2.4. The Particle Fugacities

Let $c_{ion}$, $c_{w}$, and $c_{h}$ be the bulk concentrations of ions, water, and hydrophobic particles, respectively. Let the volume fraction Φ for each type of particle be defined as:

$$\begin{array}{ccc} \Phi_{ion} & = & 2c_{ion}a^{3},\\ \Phi_{w} & = & c_{w}a^{3},\\ \Phi_{h} & = & c_{h}a^{3}. \end{array}$$

Note that we can consider a volume fraction for vacancies:

$$\Phi_{v}=1-\left(\Phi_{ion}+\Phi_{w}+\Phi_{h}\right).$$

If the system is incompressible, $\Phi_{v}=0$, and the concentrations of salt, water, and hydrophobic probes are necessarily dependent. Otherwise, $\Phi_{v}$ is positive, and vacancies are possible in the environment of the solute. In this case, $c_{s}$, $c_{w}$, and $c_{h}$ are independent, although they still need to satisfy $\Phi_{s}+\Phi_{w}+\Phi_{h}=2c_{ion}a^{3}+c_{w}a^{3}+c_{h}a^{3}\leq 1$.

The fugacities of the different particles differ if the system is considered incompressible or compressible:

(i) *Compressible system:* Vacancies are allowed and $\lambda_{v}=1$. The fugacities are given by [189]:
  $$\begin{array}{ccc} 2\lambda_{ion} & = & \frac{\Phi_{s}}{\Phi_{v}},\\ \lambda_{w} & = & \frac{\Phi_{w}}{\Phi_{v}},\\ \lambda_{h}e^{-\beta \psi_{0}} & = & \frac{\Phi_{h}}{\Phi_{v}}. \end{array}$$
(ii) *Incompressible system:* There are no vacancies among the lattice sites and $\Phi_{ion}+\Phi_{w}+\Phi_{h}=1$. The fugacities are then not independent. If we choose $\lambda_{w}=1$, the fugacities are defined as follows [189]:
  $$\begin{array}{ccc} \lambda_{w} & = & 1,\\ 2\lambda_{ion} & = & \frac{\Phi_{ion}}{\Phi_{w}},\\ \lambda_{h}e^{-\beta \psi_{0}} & = & \frac{\Phi_{h}}{\Phi_{w}}. \end{array}$$
  Finally, the bulk value $\psi_{0}$ is given by:
  $$\psi_{0}=-\frac{w_{0}}{\kappa^{2}}\frac{\Phi_{h}}{a^{3}}.$$

#### 4.2.5. The Densities or Water Dipoles, Ions, and Hydrophobic Probes

Once the fields $\varphi^{MF}\left(r\right)$ and $\psi^{MF}\left(r\right)$ have been derived as mean field solutions of the HDPBL system of equations, the densities of the various molecules defining the environment of the solute are given by the following.

(i) *Anions and cations*:
  $$\begin{array}{ccc} \rho_{\pm }\left(r\right) & = & \frac{1}{a^{3}}\frac{\Phi_{ion}e^{\mp \beta ze_{c}\varphi^{MF}\left(r\right)}}{2Z_{1}^{MF}\left(r\right)}. \end{array}$$
(ii) *Water dipoles*:
  $$\begin{array}{ccc} \rho_{w}\left(r\right) & = & \frac{1}{a^{3}}\frac{\Phi_{w}}{Z_{1}^{MF}\left(r\right)}\frac{\sinh\left(\beta p_{0}\left|\nabla \varphi^{MF}\left(r\right)\right|\right)}{\beta p_{0}\left|\nabla \varphi^{MF}\left(r\right)\right|}. \end{array}$$
(iii) *Hydrophobic particles*:

$$\begin{array}{ccc} \rho_{h}\left(r\right) & = & \frac{1}{a^{3}}\frac{\Phi_{h}}{Z_{1}^{MF}\left(r\right)}e^{-\beta (\psi^{MF}\left(r\right)-\psi_{0})}. \end{array}$$

where $Z_{1}^{MF}\left(r\right)=\Phi_{v}+\Phi_{ion}\cosh\left(\beta ze_{c}\varphi^{MF}\left(r\right)\right)+\Phi_{w}\frac{\sinh\left(\beta p_{0}\left|\nabla \varphi^{MF}\left(r\right)\right|\right)}{\beta p_{0}\left|\nabla \varphi^{MF}\left(r\right)\right|}+\Phi_{h}e^{-\beta (\psi^{MF}\left(r\right)-\psi_{0})}$.

#### 4.2.6. AquaVit

We have developed the software package AquaVit to solve the system of differential equations defining the HDPBL system. It is mostly inspired by AquaSol, a previous package developed for solving Poisson–Boltzmann-like equations [192], and uses many routines from the package MG developed by Michael Holst [193].

##### System Setup

The coordinates of the atoms of the solute as well as their partial charges are read from a single file with the PQR format. PQR files can be readily generated using the service PDB2PQR [194]. For all examples described below, we used the AMBER parameter dataset to assign charges. The PQR file is then modified to add hydrophobic “charges” to selected atoms. All atoms identified as aliphatic carbon, extended carbon, with 1, 2, or 3 hydrogens, ring carbons, and sulfur atom with one hydrogen were assigned a nonzero hydrophobic charge of +1.

The HDPBL system of equations include 12 parameters: the numbers of vertices in the mesh in each dimension, the lattice size *a*, the temperature *T*, the concentrations of water $c_{w}$, ions, $c_{s}$, and hydrophobic probes, $c_{h}$, the valence *z* of the anions and cations from the salt, the strength of the water dipole, $p_{0}$, and the parameters of the Yukawa potential, $l_{Y}\left(=\beta w_{Y}\right)$ and $l_{h}\left(=1/\kappa_{Y}\right)$. The mesh is usually set with 193 vertices in each direction. Those vertices are equally spaced, and the distance between two vertices is computed automatically based on the size of the solute and the fact that the borders of the mesh are set to be at least 14 Å away from the solute. Assuming incompressibility, in the presence of pure water, we expect $\Phi_{w}=1$, i.e., that $c_{w}a^{3}=1$, where $c_{w}$ is the concentration of bulk water, namely 55 M. This leads to a=3.11. In all the simulations described below, we have considered monovalent (i.e., z=1) ions at 0.2 M and hydrophobic particles at 1 M. As we assume incompressibility, the concentration of water is fixed and using the prescribed concentrations of ions and hydrophobic probes, and the lattice size a=3.11, we obtain the apparent concentration of water $c_{w}=53.6$ M. The parameter $l_{h}$ defines the range of the Yukawa potential; it is set to the lattice size, i.e., $l_{h}=3.1$ Å. $l_{Y}$ is a characteristic length that directly relates to its strength. We set it to $l_{Y}=4$ Å. The temperature is set to 300 K. Finally, the experimental dipole moment of water is set to 2.8D. Justifications for all those values can be found in Ref. [189].

The typical running time for characterizing the environment of a protein using AquaVit with the parameters defined above is 50 min for a grid of size $193^{3}$ on a quad-core Intel Core I7 processor running at 4.0 GHz. Ref. [189] provides a more comprehensive analysis of computing time for AquaVit on faster processors.

### 4.3. Examples

The primary goal of HDPBL is to characterize the environment around a solute of interest. In particular, it is used to detect pockets within this solute and characterize their nature, i.e., if they are more likely to accommodate a hydrophobic or a charged ligand. As such, it differs from programs that are designed to characterize the geometry of the solvent, such as UnionBall described above. We illustrate this difference on two systems, the WNV methyltransferase that accommodates a hydrophobic ligand and the WNV NS2B/NS3 protease bound to a positively charged inhibitor.

#### 4.3.1. Identifying a Hydrophobic Pocket in a Methyltransferase

WNV genomic RNA is single stranded, with positive polarity. It includes a type I cap at its 5’ end that is important for the RNA stability and translation [195]. The formation of this cap is associated with four enzymatic modifications (see for example [196]). Those modifications include the addition of a GMP at the diphosphate end, a methylation of the corresponding guanine at position N7, and a methylation of the first nucleotide of the RNA at the ribose 2’-OH position. The enzymes responsible for these modifications are encoded by the WNV genome itself. In particular, it was shown that its nonstructural protein NS5 possesses both N7 and 2’-O methyltransferase (MTase) activities (see for example [197]). The structure of this protein was determined by X-ray crystallography at 2.0 Å resolution, in the presence of sinefungin (SIN), a hydrophobic inhibitor (PDB code 3LKZ [197]).

**Figure 11:**
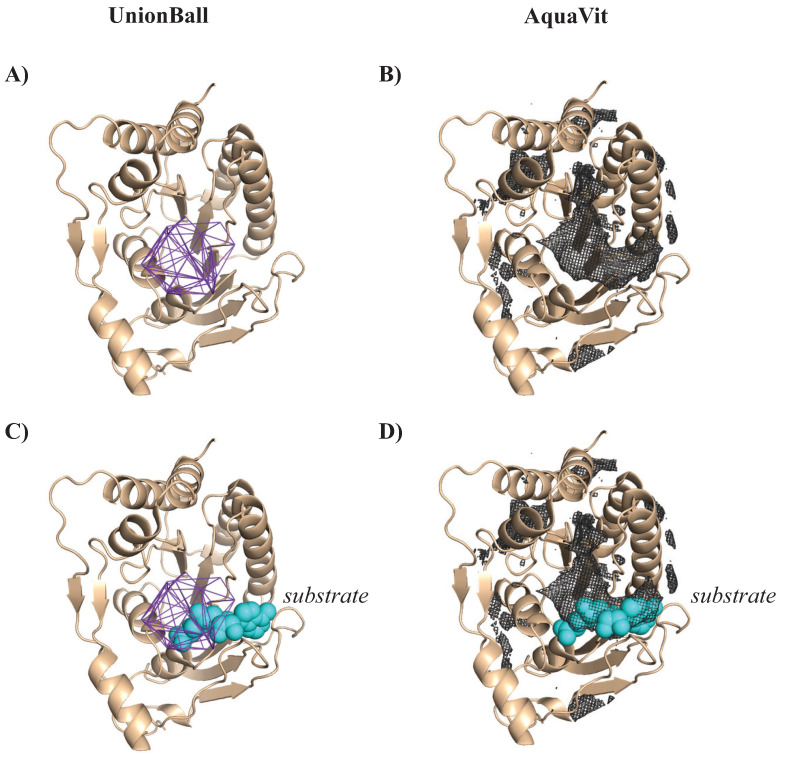
Pockets (purple) and hydrophobic occupancy maps (black mesh) superimposed onto the structure for WNV Mtase (PDB code 3LKZ). The pockets are computed with UnionBall, with a water probe of 3.0 Å. The hydrophobic maps are derived from the densities of hydrophobic particles computed by AquaVit, and represented at +20 σ. Both calculations are performed using the apo structure of the protein, i.e., in the absence of all ligands and water molecules. In panels (**A**,**B**), we show the pocket and hydrophobic maps superposed to the apo PDB structure, respectively, while in panels (**C**,**D**), we add to the figures in (**A**,**B**) the hydrophobic ligand (SIN) in cyan. Note that all images in the figure were generated using Pymol [111].

We analyzed the geometry and energetics of WNV Mtase using UnionBall and AquaVit, respectively. The PDB structure 3LKZ corresponds to this protein bound to a hydrophobic ligand, SIN. All ligands, ions, and crystallographic water molecules were removed prior to running UnionBall and AquaVit. UnionBall only includes one parameter, the radius of the water probe. We set it to 3.0 Å, to detect deep pockets within the protein. As explained above, AquaVit involves 12 parameters. Those were set as described. In Figure 11, we show the resulting pockets detected by UnionBall and hydrophobic particle occupancy map detected by AquaVit, superimposed on the PDB structure of WNV Mtase, with and without the ligand. The active site of WNK MTase is comprised of a large hydrophobic pocket, which serves as the S-adenosyl-L-methionine (AdoMet) -binding site, where AdoMet serves as a methyl donor. This pocket is conserved among flaviviruses [197]. It is successfully detected both by UnionBall and AquaVit, as illustrated in Figure 11.

#### 4.3.2. Identifying a Charged Pocket in a Protease

The replication of WNV requires a processing of its proteins by its own NS3 protease (NS3pro). NS3pro by itself, however, is virtually inactive. Its activation requires another of its protein, NS2B, as a cofactor. A high-quality structure of NS2B-NS3pro complex at a resolution of 1.68 Å in presence of the substrate-based inhibitor benzoyl-norleucine (P4)-lysine (P3)-arginine (P2)-arginine (P1)-aldehyde10 (Bz-Nle-Lys-Arg-Arg-H, BZE in short) was obtained by X-ray crystallography, available in the PDB as 2FP7 [198]. The BZE ligand is highly positively charged.

**Figure 12:**
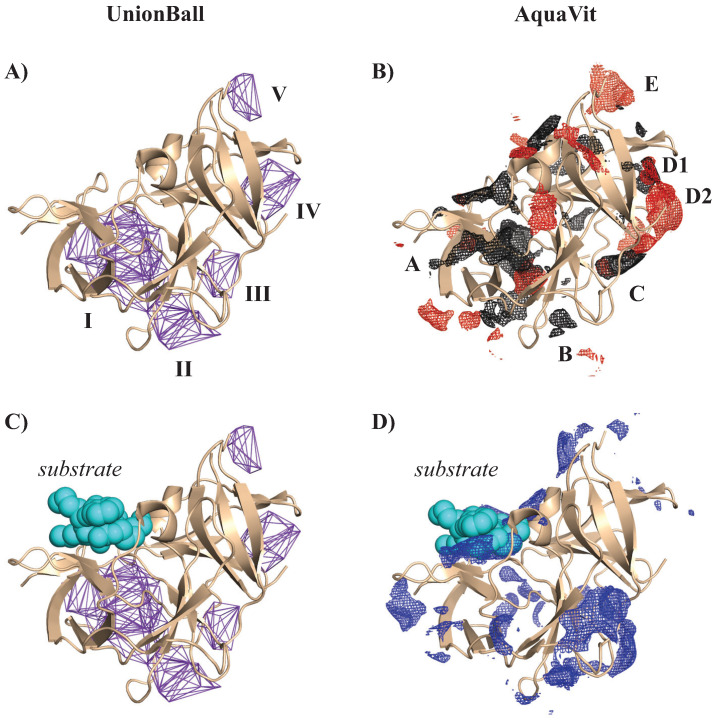
(**A**) The five largest pockets (purple) and (**B**) hydrophobic (black mesh) and anion (red mesh) occupancy maps superimposed onto the structure for WNV NS2B-NS3pro complex (PDB code 2FP7). The pockets are computed with UnionBall, with a water probe of 1.4 Å. The hydrophobic and anion maps are derived from the densities of hydrophobic particles and negative ions computed by AquaVit, and represented at +20 σ. Both calculations are performed using the apo structure of the protein, i.e., in the absence of all ligands and water molecules. Note that pockets I, II, and III identified by UnionBall match with hydrophobic pockets A, B, and C identified by AquaVit. Pocket V corresponds to the negatively charged pocket E from AquaVit, while pocket IV is a combination of a hydrophobic pocket D1 and anionic pocket D2 found by AquaVit. Panel (**C**) shows the inhibitor BEZ (see text) in cyan. The position of the inhibitor does not match with any of the five pockets found by UnionBall. Panel (**D**) shows the cation (dark blue) occupancy map superimposed onto the structure for the NS2B-NS3pro complex, with the inhibitor BEZ shown in cyan. The images in the figure were generated using Pymol [111].

We analyzed the geometry and energetics of the NS2B-NS3-pro from WNV using UnionBall and AquaVit, respectively. The PDB structure 2FP7 corresponds to the complex bound to a positively charged ligand, BEZ. All ligands, ions, and crystallographic water molecules were removed prior to running UnionBall and AquaVit. For UnionBall, the radius of the water probe was set to 1.4 Å, so that even relatively shallow pockets could be detected. The 12 parameters of AquaVit were set as described above. In Figure 12A and B, we show, respectively, the five largest pockets detecting by UnionBall and anion and hydrophobic particle occupancy maps detected by AquaVit, superimposed on the PDB structure of WNV NS2B-NS3pro complex without the ligand. Note that the pockets identified by UnionBall can be hydrophobic or negatively charged. When the ligand BEZ is superimposed to the protein complex structure and UnionBall pockets, we see that none of those pockets include the ligand (Figure 12C). The ligand sits close to the boundary between the protein and the solvent; as such, it fits in a very shallow region on the surface of the protein that cannot be detected as a pocket by UnionBall (recall Figure 1; this shallow region would correspond to one of the triangles C, D, or E that are ignored by UnionBall). In contrast, the ligand BEZ superposes well with a pocket in the cation occupancy map captured by AquaVit (Figure 12D).

## 5. Conclusions

Viruses are pathogens that raise serious threats for human health: the recent SARS-CoV-2 pandemic can unfortunately attest to this statement. Fortunately, our knowledge of those pathogens is improving. In particular, we have more and more structural information on virions (the complete, infective form of a virus that includes its genomic material and surrounding envelope) and on their gene products. This information is available in the PDB database (see, for example, Ref. [30] for a review on icosahedral virus structures in the PDB). It is important to adapt the tools and software platforms to enable the analyses of such structural information on very large macromolecular assemblies. In this paper, we have reviewed some of those methods. We focused on understanding the geometry of virions and viral structural proteins, on their dynamics, and their energetics, with the ambition that this understanding can help design antiviral agents [199]. We have discussed those methods in light of the specificities of those structures, mainly that they are very large. We focused on three of our own methods that can analyze the geometry, dynamics, and energetics of such structures within computing times that are compatible with the use of regular desktop computers. We provided (brief) explanations of the theories behind them and described the corresponding software platforms, i.e., UnionBall, NormalModes, and AquaVit. Those programs are available in open source format at the address https://www.cs.ucdavis.edu/~koehl/Projects/. We showed examples of their applications on the outer shell and some structural proteins of the West Nile Virus (WNV).

The main assumption behind all the analyses described in this review is that structural information is available on the virus that is the target for drug development. Indeed, experimental structural information on virions is becoming widely available, as mentioned above. However, it remains that such information may sometimes be still insufficient. The recent successes of AlphaFold [39] and its successor AlphaFold2 [40], two softwares that are designed to predict the structure of a protein from its sequence only, raises hope that experimental structure determination will not be a bottleneck from antiviral drug developments. The successes of using AlphaFold2 for drug discovery, however, are currently limited [44], but provide hope for better computational drug design [45].

In this review, we treated geometry, dynamics, and energetics independently: this is clearly a limitation that needs to be addressed. For example, AquaVit, our program for analyzing the environment of a protein is fast, and as such, compares favorably with the ligand mapping in molecular dynamics simulations that have been designed for detecting and characterizing binding sites in proteins. AquaVit relies, however, on the static conformation of the protein of interest, while the molecular dynamics simulations account for its dynamics. As such, they can detect cryptic binding sites in proteins [109,184,187,188], namely sites that are not accessible unless a structural change occurs. Such conformational changes are inaccessible for AquaVit. Finding ways to combine geometry, dynamics, and energetics is our future task.

## Data Availability

Not applicable.
